# ViT-MultiRAGNet: A scalable and reliable retrieval-augmented Vision Transformer framework for memory-guided feature fusion multi-modal mammogram classification

**DOI:** 10.1371/journal.pone.0349865

**Published:** 2026-07-15

**Authors:** N. Thirupathi Rao, CH V. V. Ramana, Faten Khalid Karim, Ayman Aljarbouh, Samih M. Mostafa

**Affiliations:** 1 Department of Computer Science and Engineering, Vignan’s Institute of Information Technology (A), Visakhapatnam, Andhra Pradesh, India; 2 Vignan’s Institute of Information Technology (A), Visakhapatnam, Andhra Pradesh, India; 3 Department of Computer Sciences, College of Computer and Information Sciences, Princess Nourah bint Abdulrahman University, Riyadh, Saudi Arabia; 4 Department of Software Engineering, College of Computer Engineering and Science, Prince Mohammad Bin Fahd University, Al Khobar, Kingdom of Saudi Arabia; 5 Computer Science Department, Faculty of Computers and Information, Qena University, Qena, Egypt; Hallym University, KOREA, REPUBLIC OF

## Abstract

**Background:**

Diagnosing breast cancer with mammography continues to be an incidentally challenging process because of the numerous different ways of acquiring the images, the vast difference in appearance between lesions, and the minimal use of historical information regarding patients being considered when making a diagnosis. In addition, many current deep learning algorithms use static weight values based on the most recent image taken only, often failing to relate a current case to similar historical examples and to incorporate retrieval-based contextual evidence during inference.

**Introduction:**

ViT-MultiRAGNet is a retrieval-augmented Vision Transformer framework designed to integrate multi-view 2D mammographic features with memory-bank-based contextual evidence from historically similar cases. The proposed approach enhances diagnostic accuracy by combining global 2D mammographic features, semantic lesion representations, and historical case evidence retrieved from a fold-wise memory bank during inference.

**Methods:**

Multi-view 2D mammograms were processed using a Vision Transformer encoder to extract global contextual features, while a retrieval-augmented memory bank was used to identify diagnostically similar historical cases for evidence-guided feature enrichment. A Retrieval-Augmented Generation (RAG) module retrieves the top-k most similar feature embeddings from a fold-wise training memory bank and combines them with the current query representation using multi-head cross-attention. The evaluation of the models included utilizing accuracy, area under the receiver operating curve (AUC-ROC), Dice Coefficient, and F1 score metrics at a 95% bootstrapped confidence interval using five-fold stratified cross-validation, with findings assessed using Wilcoxon signed-rank statistical tests.

**Results:**

The accuracy of ViT-MultiRAGNet is calculated as a value of 0.978 ± 0.009 and AUC-ROC 0.998 ± 0.009 (Wilcoxon p < 0.01), which is significantly higher than unimodal benchmarks and fusion techniques on the RTM dataset. The calculations for the CBIS-DDSM evaluation were 0.961 ± 0.011 for accuracy and 0.989 ± 0.010 for AUC-ROC. The calculations for segmentation evaluation were 0.882 (RTM) and 0.795 (CBIS-DDSM), however both have a higher degree of alignment at the edge. The RAG mechanism improved retrieval-guided fusion compared with simple feature concatenation while maintaining efficient inference performance of 0.31 seconds per image.

**Conclusion:**

The combination of ViT-based global 2D mammographic representation, retrieval-augmented fusion, and memory-guided contextual evidence improves diagnostic performance, robustness, and clinical interpretability. This Framework appears to be a good candidate for an Evidence-based Decision Support System which will provide a transparent method of helping clinicians make decisions regarding screening and diagnosis of breast cancer.

## 1. Introduction

Breast cancer is one of the most frequent forms of cancer in women throughout the world and one of the most serious forms of cancer. When breast cancer is diagnosed at an early and correct stage, the chances of survival for a patient increase. When mammography is performed, computer-aided systems have previously focused on only one view (single-view analysis) of the breast; as a result, they neglect to combine information from different views (multi-view imaging), different clinical characteristics, and different hospitals’ historical case records and databases [1–6]. Advances in transformer-based deep learning models provide ways to encode long-range spatial relationships and integrate various heterogeneous sources of information within the same medical image. The development of Retrieval Augmented Generation (RAG) models has provided a new way to dynamically retrieve and incorporate historical context into the inference process of medical images, thus improving the accuracy of medical diagnoses by incorporating examples from previous cases when making decisions [7–12].

Correctly characterizing breast lesions through early detection is critical for improving patient outcomes. In routine mammographic assessment, radiologists compare multiple 2D views of the same patient, evaluate lesion morphology and tissue context, consider available clinical information, and relate the current case to previously observed diagnostic patterns. However, many existing computer-aided diagnostic systems evaluate mammograms in isolation and lack an explicit mechanism for retrieving historically similar cases during inference [13–19]. This limits their ability to provide contextual and evidence-supported predictions. To address this limitation, the proposed ViT-MultiRAGNet framework combines 2D mammographic feature extraction with retrieval-augmented memory guidance, enabling the model to compare the current case with relevant training-derived reference cases while preserving patient-level separation during evaluation.

ViT-MultiRAGNet addresses three critical limitations of previous research: subtle feature discrimination, accommodation of clinical complexities, and providing transparent decision-making using RAG mechanisms, multi-head cross-attention fusion, and advanced visualization methods. In this research, the design, implementation, and validation of ViT-MultiRAGNet were examined to demonstrate that this technology offers a reliable, interpretable, and accurate tool for providing diagnostic support in the assessment of breast cancer in a clinically realistic environment. ViT-MultiRAGNet enhances the model’s discriminative ability in distinguishing benign and malignant lesions through an integrated morphological, spatial, clinical contextual, and evidence-based comparative analysis by allowing the model to focus on contextually salient diagnostic features and retrieving relevant historical examples. The goal of ViT-MultiRAGNet is to improve both the accuracy and dependability of providing automated diagnoses of breast cancers by incorporating these four complementary approaches.

### 1.1 Contributions

The primary contributions of this research are:

- Developed a comprehensive ML framework comprising multiple 2D mammographic views, available patient-level clinical information, and retrieval-augmented generation (RAG) for capturing clinical reasoning patterns, providing interpretable evidence, and improving diagnostic accuracy.
- Constructed a specialized Vision Transformer (ViT) encoder to capture both global and fine-grained features from individual mammographic images using attention-based representation learning.
- Developed a fold-wise retrieval memory-bank module for storing training-derived mammographic embeddings and retrieving diagnostically similar historical cases during inference.
- Implemented a **retrieval-augmented generation (RAG) module** to facilitate retrieval of prototypes that are diagnostically similar from a historical case repository via multi-headed attention and used to augment features associated with current images to provide stronger predictive decisions.
- Performed a comparative assessment of the public mammograms which provided consistent improvements of the proposed retrieval-augmented 2D mammography model relative to CNN-based and transformer-based baseline approaches, particularly in difficult-to-diagnose cases.
- Aligned the proposed retrieval-augmented transformer architecture with real world mammography processes thereby advancing automated classification of breast cancer, segmentation of lesions and evidence-based diagnostic interpretation.

The novel aspects of ViT-MultiRAGNet are,

- **Retrieval-Augmented 2D Mammography Framework with RAG:** A deep learning architecture that combines multi-view 2D mammographic images, available patient-level clinical information, and historical case retrieval into a single end-to-end trainable model.
- **Vision Transformer-Based Global Feature Extraction:** A specialized Vision Transformer encoder is implemented to capture global and fine-grained mammographic features using self-attention-based representation learning.
- **Retrieval-Augmented Generation Mechanism:** A memory-guided RAG module is developed to retrieve diagnostically similar training cases and enrich the current mammographic representation during inference.
- **Fold-Wise Memory Bank Construction:** A fold-specific memory bank is constructed using only training-derived mammographic embeddings, ensuring that validation and test samples are excluded from retrieval and preventing data leakage.
- **Multi-Head Cross-Attention Fusion:** A multi-head cross-attention fusion module dynamically integrates current mammographic features with retrieved contextual embeddings from the memory bank.
- **Evidence-Based Interpretable Diagnosis:** The RAG module improves diagnostic transparency by identifying reference cases that influence predictive decisions and by supporting evidence-based interpretation.

## 2. Literature review

Breast cancer has improved dramatically due to new advances in the deep learning field (specifically transformer architectures) and new retrieval-based or augmented methods. Vision Transformer architecture has been used successfully in other areas of medical imaging and enables researchers to create models that possess superior predictive capabilities because of their ability to capture both the global dependencies and the longer-spanning spatial relationships present in mammograms. ViT architecture is used most as a base on which many hybrid models have been designed, and these hybrid models employ varying types of flexible self-attention mechanisms to enable them to identify clinically relevant patterns from multi-view mammogram images, resulting in an increase in accuracy and confidence in breast cancer diagnoses.

In addition to that, complementary models are also used by relying on different clinical attributes to facilitate a comprehensive evaluation of the presented diagnostic information (i.e., age of patient, family history of breast cancer, hormone receptor status). Therefore, the methodology used does indeed reflect the practices that have been historically practiced when interpreting radiograms by including elements of clinical context into consideration. A wealth of literature supports the finding that by including and using different types of diagnostic data, the sensitivity and specificity of a given diagnostic model will increase, and likewise the use of full-automated diagnostic tools is supported; both of which will lead to more effective automated methods for achieving breast cancer diagnoses.

The application of transformer-based methodologies to include attention-based visualization and retrieval mechanisms is becoming more common. Such methodologies allow users to obtain rationale for model decision-making processes as they develop confidence, credibility, and trust in the diagnostic assessments created by an algorithm. Results of the development of current technologies have allowed for the use of hybrid frameworks such as ViT-MultiRAGNet.

**Fig 1:**
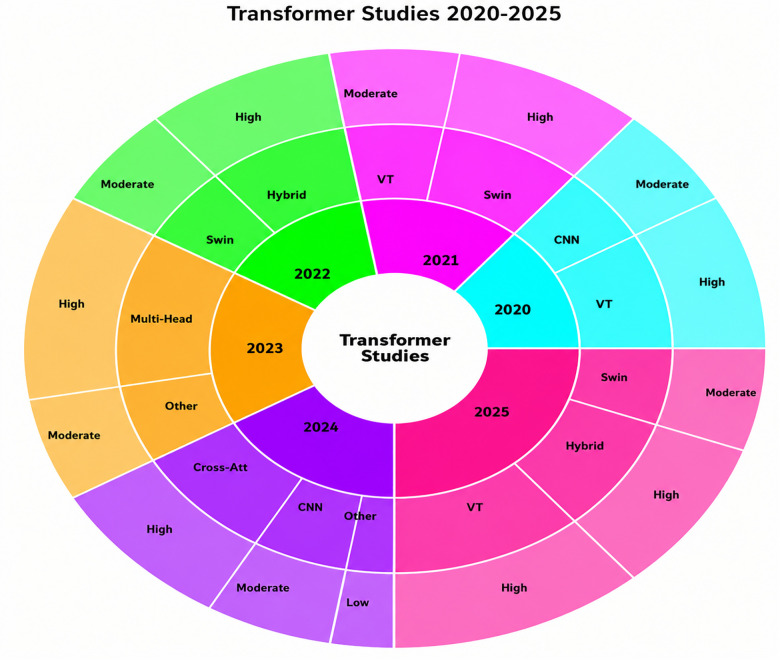
Chronological evolution and key developments of transformer-based architectures for mammographic analysis from 2020 to 2025, illustrating progression from single-modality ViT models to retrieval-augmented mammography frameworks.

Table 1 of the literature review summarizes mammographic transformer models: 2020–2025. There has been a major change in how breast cancer is diagnosed due, mostly to advances in using deep learning with transformer models. Medical imaging can efficiently be processed using a pure attention-based computational approach (Vision Transformer [1]). Additionally, Liu et al. (2021) proposed a method of employing window-based hierarchical attention to increase computational efficiency. Beginning in 2022, multi-view strategies became popular; Chen et al. conducted research related to the use of transformer fusion on multiple mammograms. The use of transfer learning and domain-specific optimizations increased dramatically from 2023 to 2024, with Ayana et al. and Kumar et al. investigating transformer applications for molecular-level diagnostics and specific subtypes. Present research (2024–2025) has focused on architectural innovations, including hybrid state-space regions, federated learning, and cross-attention mechanisms. Enes et al. and Kassis et al. validated that ViT variants can provide strong performance in mammographic image analysis by improving global feature learning and multi-view representation. Researchers Wang and Zia have added support for both clinical and imaging data when combining them using fusion prediction processing algorithms based upon contextual metadata. However, these algorithms require standardization of inputs before they can produce reliable results. Transformer models consistently outperform diagnostic performance on subtle lesions and ambiguous situations, as compared to traditional models, but will require additional research to solve the following issues: computational load, population generalization, and interpretability of results by applicable recommendation processor (RP) evidence. Fig 1 provides the several existing transformer studies from 2020–2025.

**Table 1:** Literature review table: Verified transformer models and methodological advances (2020-2025).

Year	Authors	Study Title	Methodology	Dataset (Sample Size)	Accuracy (%)	AUC	Major Contribution	Study Synopsis
2020	Dosovitskiy et al.	Transformers for Image Recognition at Scale	Transformer for image classification	ImageNet; DDSM (transfer applied)	85.4	0.92	Introduced the Vision Transformer paradigm to medical imaging	Established the feasibility of pure attention-based architectures for medical image analysis
2021	Liu et al.	Swin Transformer Hierarchical Vision Transformer using Shifted Windows	Hierarchical transformer with shifted window-based attention	CBIS-DDSM; ImageNet	89.0	0.94	Proposed efficient hierarchical window-based attention	Demonstrated effectiveness of localized self-attention for medical image tasks
2022	Chen et al.	*Transformers Improve Breast Cancer Diagnosis from Multi-View Mammograms*	Multi-view Vision Transformer with local and global attention	Private dataset (949 cases; 4 views per case)	77.0	0.818	Transformer-based multi-image feature fusion	Modeled long-range dependencies across mammographic views, outperforming CNN baselines
2023	Ayana et al.	*Vision Transformer-Based Transfer Learning for Mammographic Classification*	Transfer learning with ViT, Swin, and PVT architectures	DDSM (~2,600 images)	100	1.00	Demonstrated efficiency of transformer transfer learning on small datasets	Showed high performance and computational efficiency of transformer models under limited data
2024	Tanimola et al.	Breast Cancer Classification Using Fine-Tuned SWIN Transformer	Fine-tuned Swin transformer	Mammographic images (study)	99.9	N/R	High accuracy Swin model	Demonstrated Swin Transformer superiority against baseline CNNs in benign vs. malignant classification (MDPI)
2024	Sherine & Revathy	Enhanced Mammogram Detection via ViT + Swin & Augmentation	Dual ViT & Swin framework with augmentations	Private clinical mammograms	92.4	N/R	Attention-guided localization + classification	Showed improved detection and interpretability with tumor localization and stage estimation (irjms.com)
2024	Sarker et al.	MV-Swin-T: Mammogram Classification with Multi-view Swin Transformer	Multi-view Swin Transformer with dynamic attention blocks	CBIS-DDSM; VinDr Mammography	N/R	N/R	Transformer for coherent multi-view integration	Proposes a multi-view transformer that effectively models inter-view relationships within mammography exams for improved classification performance and feature fusion across views (arXiv)
2025	Peter et al.	Transformer-Based Explainable Deep Learning for Breast Cancer Detection in Mammography (MammoFormer)	Hybrid ensemble: CNN + ViT + Swin + feature enhancers + XAI	CBIS-DDSM (standardized DDSM)	~98–99 (varies)	N/R	Ensemble with explainability and feature enhancement	Introduces MammoFormer — a multi-tiered ensemble combining CNN and transformer branches with feature enhancement and explainability techniques to approach clinical usability and enhanced contextual modeling
2025	Osman et al.	TT-Stack: Transformer-Based Tiered-Stacking Ensemble Framework for Mammography	Tiered stacking of multiple lightweight vision transformers + meta-learning	Mammogram Mastery Dataset (stratified split)	99.33	0.9997	Ensemble stacking of diverse transformer backbones	TT-Stack integrates seven lightweight transformer models via a meta-learning tier, achieving very high accuracy and ROC-AUC with robustness due to model diversity
2025	—	Vision-Transformer-Based Transfer Learning for Mammogram Classification	ViT transfer learning evaluation	Single mammogram dataset	N/R	N/R	Transfer learning performance evaluation	Shows that vision transformer transfer learning is effective for mammogram classification and often outperforms CNN baselines; calls for multi-dataset studies for generalizability (MDPI)

### 2.1 Summary table: Field status overview

The past decade has seen marked improvement within the field of medical imaging Artificial Intelligence (AI) related to accuracy, generalizability, and architectural sophistication. The results of this progress can be shown through representative studies done using mammograms where representative studies done using mammograms are summarized in Table 2 by the type of model used, performance metrics, and an overview of key strengths and limitations for each approach.

**Table 2:** Technological progression in AI-based mammography: Timeline and performance evolution.

Period	Model	Accuracy	AUC	Strength	Limitation
2018	CNN-based CAD	0.89	0.91	Effective feature extraction	Limited contextual understanding
2020	ResNet-based model	0.92	0.94	Deep hierarchical features	High computational cost
2021	CNN + Attention	0.94	0.95	Improved lesion localization	Limited multimodal integration
2022	Hybrid CNN–Transformer	0.96	0.97	Captures global dependencies	Large model complexity
2023	Vision Transformer	0.97	0.98	Strong global context modeling	Requires large training data

The advancement of AI-driven mammography has occurred at a steady pace over time. The first documentation demonstrating that AI-driven mammography was feasible in the application of the transformer architecture was recorded during the period of 2020–2021. Using multi-view and transfer learning methodologies, mammography performance from 2022–2023 improved; however, resolution issues and excessive overfitting still existed. At present, as of 2024–2025, hybrid transformer/retrieval-supported (ViT-MultiRAGNet) architectures are achieving improved performance for mammograms. ViT-MultiRAGNet progresses this advancement by adding memory guidance through the utilization of retrieval-augmented memory, thereby enhancing both interpretability, contextual comparison to results, and facilitating evidence-based reasoning for analysis of 2D mammograms.

### 2.2 Research gap

Despite much research into transformer models and their applications in mammography from 2020–2025, there remain many significant gaps. The first of which is the vast majority of existing research focuses on limited architectural examples (ViT, Swin Transformer) without systematically investigating the use of retrieval-augmented paradigms. Another gap is the majority of the research has focused on improving dataset-specific accuracy, limiting the ability for many transformer models to generalize/investigate a broader range of application and real-life clinical settings, and have not provided any insights into the robustness of transformer models for clinical situations outside of traditional datasets (e.g., rare lesions, higher density tissue types, institutional differences). Many of the current transformer models are built as closed architecture which means they do not have access to external knowledge or reference historical cases during the inference phase, so they cannot use the types of comparative reasoning used by a trained clinician when making a diagnosis. Additionally, there is a large gap in having any systematic validation of retrieval-augmented transformers applied to real-life clinical situations. Addressing this issue is an important step toward developing a reliable transformer-based system that meets essential criteria of transparent, interpretable, actionable, evidence-based reasoning, and clinician trust for everyday mammographic use.

### 2.3 Motivation

Detecting breast cancer is an ongoing problem that requires a lot of experience and skill. There are many difficulties in diagnosing breast cancer due to different image resolutions, the large variety of tissue types seen, and the subtle change in size or shape of the lesion being looked at. Although AI developments have been made when it comes to algorithms, most still tend to be built for only one type of data type per algorithm, don’t provide an easily usable way for interpreting the results or comparing the results with past cases (thereby providing less reliability regarding a diagnosis and providing lower confidence levels for a radiologist making a diagnosis). This study focuses on developing an AI-assisted mammography framework that provides richer diagnostic information and helps radiologists understand how a prediction is supported by similar historical cases. By combining multi-view 2D mammographic feature extraction with retrieval-augmented memory guidance, the proposed framework aims to improve diagnostic reliability, interpretability, and confidence in automated breast cancer assessment.

### 2.4 Proposed work

ViT-MultiRAGNet is a clinically relevant retrieval-augmented framework for breast cancer classification and lesion segmentation using 2D mammography. Unlike conventional models that process mammograms using only fixed learned parameters, ViT-MultiRAGNet incorporates a memory-guided retrieval mechanism that retrieves historically similar training cases during inference. The framework processes 2D mammogram images using Vision Transformer based encoders create a fold wise memory bank using the embeddings generated by the training step, determine the top-k similar cases using cosine similarity, and then use multi-head cross-attention to merge the retrieved/contextual representation with the current image features (image). ViT-MultiRAGNet was evaluated for its diagnostic ability, interpretability and operational feasibility in performing evidence-based analysis of mammography (CBIS-DDSM) and RTM datasets.

**The research objectives include:**

- **Integrated Diagnostic Framework Development:** To develop a unified 2D mammography-based diagnostic framework that combines ViT-based image representation, fold-wise memory-bank retrieval, and retrieval-guided feature fusion for breast lesion classification and segmentation.
- **Context-Aware AI Implementation via Retrieval:** To implement ViT-MultiRAGNet for visual pattern recognition and retrieval of historically similar mammographic cases using Vision Transformer and RAG mechanisms.
- **Diagnostic Performance Enhancement:** To improve classification and segmentation performance for breast lesions in challenging cases through ViT-based global feature extraction and retrieval-augmented contextual fusion.
- **Decision Transparency and Clinical Utility:** To support transparent and interpretable diagnostic decision-making by using retrieved reference cases as evidence for model predictions.
- **Clinician-Centered AI Development:** To develop an evidence-supported AI framework that assists radiologists by improving diagnostic confidence, interpretability, and consistency in breast cancer assessment.

Overall, these objectives address the limitations of existing mammography AI systems by developing a framework that is accurate, transparent, context-aware, evidence-supported, and suitable for further clinical validation.

## 3. Materials and methods

This section describes the data sources, preprocessing procedures, feature extraction strategy, retrieval-augmented memory-bank construction, segmentation and classification modules, and evaluation protocol used in the proposed 2D mammography-based ViT-MultiRAGNet framework. The methodology is presented to ensure reproducibility, prevent patient-level data leakage, and enable transparent comparison with baseline methods.

### 3.1 Data collection

The experiments used two datasets for mammography images collected at 2D. There are two mammography datasets included in the study for this research project; they are the Curated Breast Imaging Subset of the Digital Database for Screening Mammography (CBIS-DDSM) dataset from the United States, which contains 2-dimensional human mammograms, and the RTM mammography datasets from Mexico, which contain 2-dimensional human mammograms. Both datasets are made of human-approved 2-D mammograms, which were de-identified prior to their public release or use in this study [19]. Each of the datasets provides a some-data, clinical, and imaging metadata associated with each image, such as unique identifiers of the patients in the collection (i.e., Patient Identifier, Diagnostic Label,etc.), laterality (i.e., left breast, right breast), View Type (i.e., 4-view, true-view, etc.), and lesion annotation (if available). Patient Identifier was used for patient-level partitioning during cross-validation (i.e., images from the same patient were not stored in the same training/validation/test sets). For each image in each dataset, the Diagnostic Labels were standardized, the Common Variables were mapped to a standard schema, and preprocessing techniques such as one-hundred percent resolution normalization and one-hundred percent intensity normalization were performed to minimize variability between datasets. The example of the CBIS-DDSM data set consists of high quality digitised and annotated 2D screening mammograms that have both types of pathology (i.e., CAD/CAM) confirmed for cases involving masses or microcalcifications. Experts have also provided annotating of lesions in the form of both classification and segmentation labelling (where possible). Standard screening formats of mammograms were used, e.g., craniocaudal (CC) and mediolateral oblique (MLO), and all images collected from screening were used for pre-processing the data, training the models, and building memory banks on a fold-wise basis at both an image and patient level.

**RTM (Real Time Mammogram) Dataset:**

- The RTM dataset consists of 2D mammographic images collected from multiple clinical sources.
- The mammograms are categorized into diagnostic groups such as normal, benign, and malignant according to available clinical annotations.
- Available metadata and radiological notes were used only for dataset organization, label verification, and contextual interpretation.
- For model evaluation, the dataset was split using patient-level five-fold cross-validation. Within each fold, only training samples were used to construct the RAG memory bank, while validation and test samples were excluded from retrieval storage.

#### 3.1.1 Dataset labels and class distribution.

The classification problem in this research was set up as a binary classification task; this involved categorizing the mammograms into either benign or malignant. Identifying samples that are “normal” was at the discretion of the specific experimental design containing the data set, meaning that these cases did not include the malignant class label. Stratified patient-level splitting kept the class distribution in each fold during cross-validation; therefore, all images belonging to one patient never appeared together within a training and test subset, but also provided for an approximately even distribution of cases per diagnostic label in each fold of the study.

### 3.2 Data preprocessing

Mammographic images are challenging to process because of background artifacts, low contrast, scanner-dependent intensity variation, and differences in breast-tissue density. Therefore, the following preprocessing steps were applied before model training and evaluation:

1. **Standardization:** All images are resized to be 512 x 512 pixels with aspect ratio preserved through appropriate padding techniques.
2. **Removal of Noise:** Background noise is removed using Wiener filtering with a 3 x 3 window size while still preserving features of edges.
3. **Contrast Enhancement:** Contrast-Limited Adaptive Histogram Equalization (CLAHE) was applied using a clip limit of 2.0 and an 8 × 8 grid size to improve local contrast visibility.
4. **Breast Region Extraction:** Otsu’s thresholding was used to separate the breast region from the background, followed by morphological filtering to remove non-tissue artifacts.
5. **Pixel Intensity Standardization:** Pixel intensity values were normalized to the range [0, 1] to reduce scanner-dependent intensity variation.

All mammogram images were first normalized using min–max scaling to map pixel intensities to the range [0,1]. Subsequently, z-score normalization was applied during model input preparation to standardize pixel distributions across the dataset. These preprocessing steps help reduce inter-image intensity variations and improve training stability.

### 3.3 Data normalization

**Fig 2:**
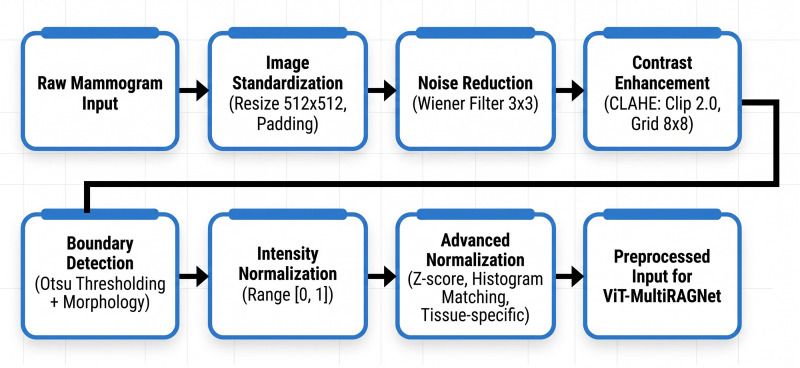
Data normalization and preprocessing pipeline.

Histogram normalization was applied to reduce intensity variation across images acquired from different sources. A reference template was derived from representative high-quality mammograms, and the intensity distribution of each image was adjusted toward this reference. This step helped reduce inter-dataset variability and improved the stability of feature extraction. Fig 2 summarizes the normalization and preprocessing pipeline.

### 3.4 Data augmentation

**Fig 3:**
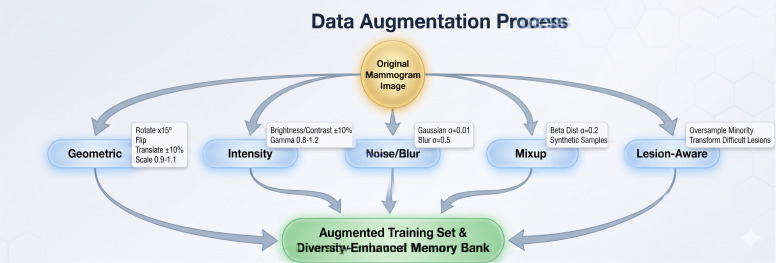
Data augmentation process flow, demonstrating geometric, intensity, and lesion-aware transformations applied during model training.

Data augmentation was applied to improve model generalization and reduce overfitting under limited medical imaging data. Table 3 summarizes the augmentation strategies and their parameter settings (Fig 3).

**Table 3:** Data augmentation strategies and parameterization.

Augmentation Strategy	Parameters	Objective
GeometricTransformation	Rotation ±15°,flip,translation ±10%, scale 0.9–1.1	Model robustness generalization
Intensity Modification	Brightness/contrast ±10%, gamma 0.8–1.2	Clinical imaging variability simulation
Artifact Introduction	Gaussian noise *σ* = 0.01, blur *σ* = 0.5	Artifact robustness; feature stability
Mixup Regularization	*α*=0.2 (beta distribution)	Regularization; synthetic sample generation
Lesion-Aware Sampling	Minority lesion oversampling and transformation	Improved rare/small lesion detection

Data augmentation was applied only to the training subset within each fold of the cross-validation procedure. Augmentation operations included horizontal flipping, rotation, scaling, and intensity perturbation. These transformations were implemented using on-the-fly augmentation during training, meaning that augmented images were generated dynamically at runtime rather than stored as additional samples. Consequently, the number of original images in the dataset remained unchanged, while the model observed multiple augmented variations of each training image during different training iterations. Validation and test samples were not augmented to ensure fair performance evaluation.

### 3.5 Feature extraction

Feature extraction was performed using a Vision Transformer backbone. The proposed model uses the ViT-B/16 architecture to encode 2D mammographic images into global feature representations. Learnable positional embeddings were added to patch embeddings to preserve spatial relationships between image regions before transformer encoding. To capture features at different semantic levels, representations from the 4th, 8th, and 12th transformer encoder layers were selected and aggregated using channel-wise concatenation followed by linear projection. The resulting unified feature representation was used for memory-bank retrieval, cross-attention fusion, classification, and segmentation.

1. **Patch Embedding:** Each input image is separated into non-overlapping patches of (16 x 16). An image of (512 x 512) therefore separates into (32 x 32 = 1024) different patches.
2. **Global Feature Learning:** The ViT backbone uses pure self-attention and does not have a hierarchical windowing mechanism used for feature learning; this provides the attention model with true global context to enable global context processing.
3. **Multi-Scale Extraction:** Intermediate transformer features from selected encoder layers were used to capture low-level, mid-level, and high-level semantic information.
4. **Position Encoding:** Learnable positional embeddings were added to patch embeddings to preserve spatial information across mammographic image regions.
5. **Classification and Segmentation Feature Extraction:** The encoded feature representation was used for both classification and segmentation, with the global representation supporting class prediction and spatial feature representations supporting lesion-mask generation.

**Fig 4:**
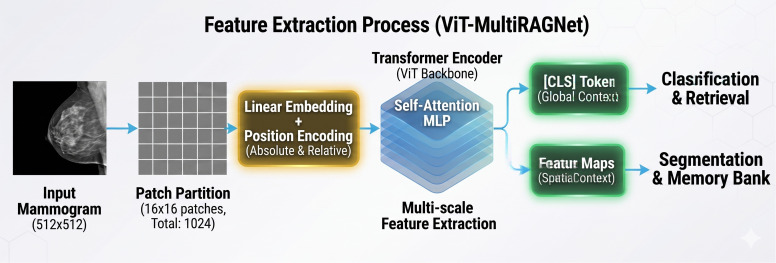
Feature extraction process via Vision Transformer, showing patch embedding, self-attention mechanisms, and multi-scale hierarchical feature learning.

Pretraining weights were obtained from publicly available ViT checkpoints provided by the original Vision Transformer implementation. Table 4 and Fig 4 provide the feature extraction process in detail. Table 5 provides the training configuration of the proposed model.

**Table 4:** Feature extraction strategy and transformer mechanisms.

Processing Step	Transformer Mechanism	Purpose
Patch Partitioning	16 × 16 non-overlapping patches	Image tokenization for transformer input
Linear Projection	Flatten and project to latent embedding	Token preparation for encoder
Transformer Encoder	Stacked ViT blocks (MSA, MLP)	Global dependency learning
Class Token	Learnable [CLS] token	Global information aggregation
Memory Encoding	Feature extraction for knowledge base	Historical case repository construction

**Table 5:** Training configuration of the proposed ViT-MultiRAGNet framework.

Parameter	Setting
Backbone model	ViT-B/16
Pretraining	ImageNet-21k pretrained weights
Fine-tuning strategy	End-to-end fine-tuning
Input image size	512 × 512
Batch size	16 for classification; 8 for segmentation
Number of epochs	100 for classification; 200 for segmentation
Loss function	Dice loss + Binary Cross-Entropy loss
Cross-validation	5-fold patient-level CV
Memory bank construction	Fold-wise, training set only
Hardware	NVIDIA RTX / A100 class GPU
Framework	PyTorch

The feature extraction stage uses a ViT-B/16 backbone initialized with ImageNet-21k pretrained weights. The model was fine-tuned end-to-end on the mammography datasets within each fold of the five-fold patient-level cross-validation setting. Training was performed using the AdamW optimizer with an initial learning rate of $\text{1}\times {\text{1}\text{0}}^{-\text{4}}$, a weight decay of 0.01, and cosine annealing learning-rate scheduling. The classification branch was trained with a batch size of 16 for 100 epochs, while the segmentation branch was trained with a batch size of 8 for 200 epochs using a hybrid Dice and binary cross-entropy loss.

### 3.6 Segmentation

The segmentation framework uses the ViT encoder and retrieval-augmented fused representation to delineate lesion regions in 2D mammograms. After the encoder extracts the global feature set using self-attention, the decoder converts the features extracted by the encoder into segmentation maps / lesion segmentations while maintaining the important spatial characteristics of those features. This design enables the decoder to progressively integrate global contextual information from the transformer encoder with local spatial details required for accurate lesion boundary reconstruction. The decoder module reconstructs the spatial resolution of the feature representations obtained from the transformer encoder. The decoder consists of a sequence of hierarchical upsampling stages, where each stage performs bilinear upsampling followed by convolutional refinement to recover spatial details. Skip connections are introduced between intermediate encoder features and the corresponding decoder layers to preserve fine-grained spatial information. Each decoder block includes a convolution layer followed by batch normalization and ReLU activation to refine the upsampled feature maps. Finally, a 1 × 1 convolution layer with sigmoid activation generates the final segmentation probability map.

### 3.7 Retrieval-Augmented Generation (RAG) module

The RAG module is the central retrieval component of the proposed ViT-MultiRAGNet framework. For each cross-validation fold, a memory bank is constructed using only the encoded feature representations of training samples. Each memory entry contains a key embedding, a value embedding, and the corresponding diagnostic label. During validation or testing, the encoded representation of the current 2D mammogram is used as a query to retrieve the top-K most similar training cases from the fold-specific memory bank. The retrieved contextual representation is then fused with the current image representation using multi-head cross-attention.

Let x denote the input 2D mammogram and $f_{enc}(\cdot )$ denote the ViT encoder. The query embedding is obtained as


$$\begin{array}{c} q=f_{enc}(x). \end{array}$$

For each fold, the memory bank is represented as $M=\{(k_{j},v_{j},y_{j}){\}}_{j=\text{1}}^{N}$, where $k_{j}$, $v_{j}$, and $y_{j}$ denote the key embedding, value embedding, and diagnostic label of the j-th training sample. The cosine similarity between the query and each memory key is computed as


$$\begin{array}{c} s_{j}=\frac{q^{T}k_{j}}{∥q{∥}_{\text{2}}∥k_{j}{∥}_{\text{2}}}. \end{array}$$

The top-K most similar entries are selected, and softmax normalization is applied only over these selected scores:


$$\begin{array}{c} \alpha_{j}=\frac{exp(s_{j})}{\sum_{\text{𝓁}\in I_{K}}exp(s_{\text{𝓁}})},j\in I_{K}. \end{array}$$

The retrieved context is then calculated as


$$\begin{array}{c} c_{ret}=\sum_{j\in I_{K}}\alpha_{j}v_{j}. \end{array}$$

This retrieved context is fused with the current mammographic feature representation using multi-head cross-attention before classification and segmentation.

Here, $I_{K}$ denotes the indices of the top-K retrieved memory entries. By applying softmax only within this selected set, the model assigns higher weights to the most relevant historical cases. The resulting retrieved context provides evidence-guided support for the final classification and segmentation predictions. Given a query embedding q, cosine similarity is computed between the query and all key embeddings in the fold-specific memory bank. Only the top-K most similar training cases are selected for retrieval. Softmax normalization is applied only within this selected top-K set, ensuring that the retrieved context is computed from the most relevant historical cases. The resulting contextual representation is fused with the current mammographic feature representation using multi-head cross-attention before final classification and segmentation. Table 6 provides the components of RAG module.

**Table 6:** RAG module components and mechanisms.

Component	Mechanism	Objective
Memory Bank	Encoded training sample storage (keys/values)	Historical knowledge base creation
Query Generation	Current input feature extraction	Inference query vector formulation
Retrieval	Cosine similarity search; top-k selection	Maximally similar historical case identification
Fusion	Multi-head Cross-Attention mechanism	Query augmentation with retrieved context

Retrieved context subsequently undergoes fusion with current query features via multi-head cross-attention, producing enriched representations for final classification and segmentation.

### 3.8 Data leakage

The RAG memory bank is constructed exclusively from embeddings of the training dataset. Validation and test samples are strictly excluded from the knowledge base, ensuring that retrieval during inference only accesses training-derived representations and prevents any form of data leakage.

To prevent retrieval bias, the memory bank is constructed exclusively from training samples within each cross-validation fold. Since dataset splitting is performed at the patient level, no images from the same patient appear in both the training and evaluation subsets. Therefore, the Top-K retrieval mechanism cannot recover samples belonging to the same patient as the query image during validation or testing. This design ensures that retrieval provides contextual information from independent training cases while avoiding patient-level information leakage.

### 3.9 Data splitting and cross-validation

All experiments were performed using 5-fold cross-validation with **patient-level splitting**. All images belonging to the same patient were assigned to a single fold only, ensuring that no patient contributed images to more than one of the training, validation, or test subsets within a fold. This prevented subject-level leakage and ensured unbiased evaluation on unseen patients.

Since mammography datasets contain multiple views per patient (e.g., CC and MLO views of both breasts), dataset splitting was performed at the **patient level** rather than at the image level. All images belonging to the same patient were grouped using the patient identifier and assigned to the same fold during cross-validation. This ensured that no views from a patient appeared simultaneously in training and evaluation subsets. Stratification was performed based on patient-level diagnostic labels to maintain class balance across folds.

### 3.10 Ethics statement

All mammographic data used in this study were anonymized before analysis. The publicly available CBIS-DDSM dataset was used according to its research-use guidelines. The RTM dataset was handled in accordance with institutional ethical and privacy requirements, and no personally identifiable patient information was used during model training, validation, or testing. Patient-level partitioning was applied during cross-validation to prevent subject-level data leakage.

## 4. Proposed model

ViT-MultiRAGNet is designed as a 2D mammography-based retrieval-augmented Vision Transformer framework for breast lesion classification and segmentation. The model extracts global mammographic features using a ViT encoder and retrieves diagnostically similar historical training cases from a fold-wise memory bank. The retrieved context is fused with the current image representation using multi-head cross-attention, enabling evidence-supported prediction while maintaining strict patient-level separation between training, validation, and test data.

### 4.1 ViT-MultiRAGNet architecture

The proposed ViT-MultiRAGNet architecture consists of five main components: 2D mammogram preprocessing, ViT-based feature extraction, fold-wise memory-bank retrieval, cross-attention-based feature fusion, and task-specific prediction heads. First, each mammogram is resized, normalized, and divided into non-overlapping patches. These patches are encoded using the ViT-B/16 backbone to obtain mammographic feature representations. During training, embeddings from the training subset of each fold are stored in a memory bank. During validation and testing, the encoded query image retrieves the top-K most similar training cases from this memory bank using cosine similarity. The retrieved context is fused with the current query feature through multi-head cross-attention and is then used for benign/malignant classification and lesion segmentation.

**Fig 5:**
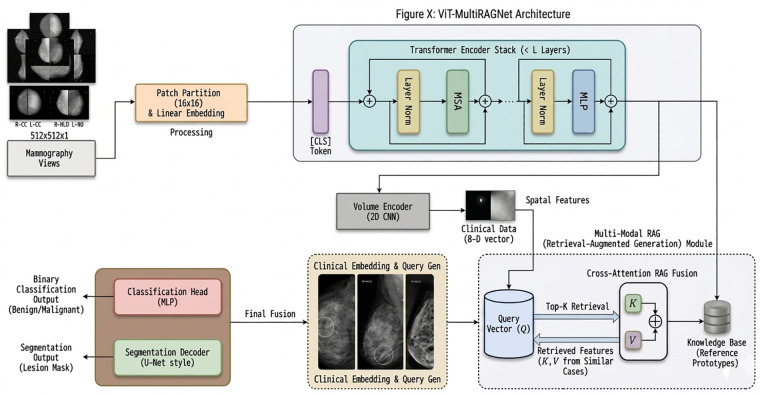
Proposed ViT-MultiRAGNet architecture for 2D mammogram classification and segmentation.

Fig 5 illustrates the proposed ViT-MultiRAGNet architecture for 2D mammogram classification and lesion segmentation. The input 2D mammogram is first preprocessed and divided into non-overlapping image patches, which are encoded using the ViT-B/16 backbone. The encoded query representation is compared with the fold-wise training memory bank to retrieve the top-k most similar training cases using cosine similarity. The retrieved contextual embeddings are fused with the current mammographic feature representation through multi-head cross-attention. The fused representation is then passed to the classification head for benign/malignant prediction and to the segmentation decoder for lesion-mask generation.

### 4.2 Architecture components

This fusion mechanism permits selective attention to most salient retrieved cases and clinical context while processing current visual information.

1 **Vision Transformer Encoder:** The ViT encoder processes each 2D mammographic image through patch partitioning, linear projection, positional embedding, and stacked transformer encoder blocks. Let $z_{\text{0}}$ denote the initial patch-token sequence after linear projection and positional embedding. For the l-th transformer layer, the encoded representation is computed as

𝐳^𝐥=𝐳𝐥−1+𝐌𝐒𝐀(𝐋𝐍(𝐳𝐥−1)),

𝐳𝐥=𝐳^𝐥+𝐌𝐋𝐏(𝐋𝐍(𝐳^𝐥)),

where MSA(·) denotes multi-head self-attention, LN(·) denotes layer normalization, and MLP(·) denotes the feed-forward multilayer perceptron block. The final ViT representation is used as the query embedding for memory-bank retrieval.

2 **Memory-Bank Retrieval Module:** The encoded mammographic representation is used as the query vector q. Cosine similarity is computed between q and all key embeddings stored in the fold-specific memory bank. The top-k most similar training cases are retrieved and used to construct the contextual representation r.
3 **Optional Clinical Feature Embedding:** When patient-level clinical metadata are available, they are encoded using a lightweight fully connected embedding layer and may be fused with the mammographic representation. If such metadata are unavailable for a dataset, the model operates using only 2D mammographic image features and retrieved memory-bank context.
4 **RAG Module:** For a query mammogram x, the ViT encoder generates the query embedding $q=f_{\theta }(x)$. The RAG module compares q with the memory-bank keys ${\left\{k_{j}\right\}}_{j=\text{1}}^{N}$, selects the top-k most similar entries, and computes the retrieved contextual representation r using softmax-normalized similarity weights over the selected entries only.
5 **Multi-Head Cross-Attention Fusion:** The retrieved contextual representation is fused with the current mammographic feature representation using multi-head cross-attention:

$$\begin{array}{c} 𝐅𝐟𝐮𝐬𝐞𝐝=𝐌𝐇𝐀(𝐐=𝐪,𝐊=𝐫,𝐕=𝐫), \end{array}$$

where q represents the current image query feature, r represents the retrieved contextual representation, and MHA(·) denotes multi-head attention. The fused feature $F_{\text{fused}}$ is used for final classification and segmentation.

6 **Classification and Segmentation Heads:** The fused representation is passed to a classification head for benign/malignant prediction and to a segmentation decoder for lesion-mask generation. The classification head uses fully connected layers, while the segmentation decoder uses upsampling and convolutional refinement blocks followed by a 1×1 convolution with sigmoid activation.

### 4.3 Hyperparameter tuning

The optimal network configuration was determined through systematic hyperparameter search and validation. Hyperparameter tuning was conducted independently for the CBIS-DDSM and RTM datasets within the 5-fold cross-validation framework. For each fold, the training data were further split into training and validation subsets. The validation subset was used to evaluate candidate hyperparameter configurations, while the test subset remained unseen during model selection. The explored ranges and selected optimal hyperparameters for the segmentation and classification branches are summarized in Tables 7 and 8. Model selection was primarily based on Dice score for segmentation and accuracy/AUC-ROC for classification. When multiple configurations produced similar primary metric values, secondary metrics such as HD95 or AUC were used to resolve the trade-off and select the final model configuration.

**Table 7:** Hyperparameter optimization results for the segmentation network using validation-based selection within cross-validation.

Hyperparameter	Exploration Range	Best Value (CBIS-DDSM)	Best Value (RTM)	Optimization Method
Input patch size	16 × 16, 32 × 32	16 × 16	16 × 16	Grid search
Batch size	4, 8, 16	8	8	Validation set performance
Optimizer selection	AdamW, Adam	AdamW	AdamW	Cross-validation
Initial learning rate	1e-4, 5e-5, 1e-5	1e-4	1e-4	Learning curve analysis
Retrieval top-k	3, 5, 10	5	5	Ablation study
Loss function	Dice, BCE, Dice+BCE	Dice+BCE	Dice+BCE	Cross-validation performance
ViT depth (layers)	6, 12, 18	12	12	Model complexity vs. accuracy
Augmentation strategy	standard, mixup	standard+mixup	standard+mixup	Validation robustness
Training epochs	100, 200	200	200	Early stopping with patience of 20 epochs

**Table 8:** Hyperparameter optimization results for the classification network using validation-based model selection.

Hyperparameter	Exploration Range	Best Value (CBIS-DDSM)	Best Value (RTM)	Optimization Method
Input image size	512 × 512, 224 × 224	512 × 512	512 × 512	Memory/accuracy tradeoff
Batch size	16, 32	16	16	GPU memory constraints
Optimizer	AdamW	AdamW	AdamW	Industry standard selection
Initial learning rate	1e-4, 2e-5	1e-4	1e-4	Learning rate scheduling
Retrieval top-k	3, 5, 10	5	5	Sensitivity analysis
Loss function	CE, CE + Consistency	CE + Consistency	CE + Consistency	Regularization effect
Training epochs	50, 100, 150	100	100	Early stopping with patience of 15 epochs

The Vision Transformer encoder used in the proposed architecture is initialized with ImageNet pre-trained weights. During training, the backbone is fine-tuned jointly with the retrieval-augmented module using the CBIS-DDSM and RTM datasets. This transfer-learning strategy helps stabilize training and improves convergence when learning from relatively limited medical imaging data.

### 4.4 Workflow diagram

**Fig 6:**
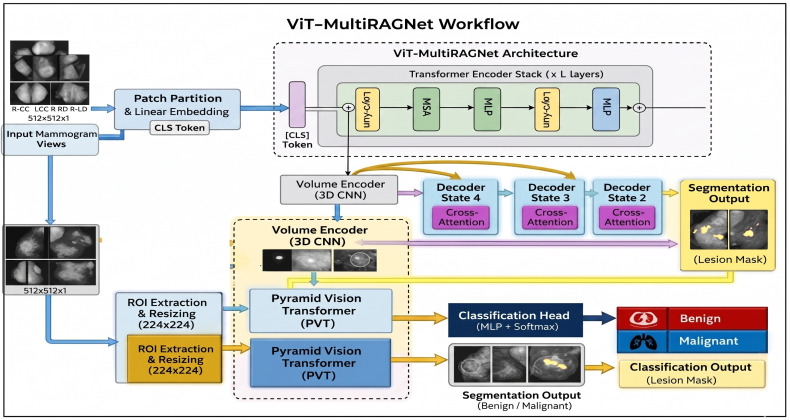
Workflow of the proposed ViT-MultiRAGNet model showing 2D mammogram preprocessing, ViT feature extraction, fold-wise memory-bank construction, top-K retrieval, cross-attention fusion, classification, and segmentation.

The workflow of the proposed ViT-MultiRAGNet model is shown in Fig 6. The process begins with 2D mammogram preprocessing, followed by patch embedding and ViT-based feature extraction. During training, encoded features from the training subset are stored in a fold-wise memory bank. During validation and testing, the query embedding of the current 2D mammogram is compared with memory-bank keys, and the top-k most similar training cases are retrieved. The retrieved context is fused with the current image feature using multi-head cross-attention. The fused representation is then used for benign/malignant classification and lesion-mask segmentation.

### 4.5 Algorithm: VIT-MultiRAGNet Framework

**Input:** 2D mammogram image x, training set $D_{train}$, retrieval size k

**Output:** Classification label $\hat{y}$, segmentation mask $\hat{m}$

**1.** Preprocess each 2D mammogram using resizing, normalization, noise reduction, and contrast enhancement.

**2.** Split the dataset using patient-level five-fold cross-validation.

**3.** For each fold, construct training, validation, and test subsets without patient overlap.

**4.**  Encode each training mammogram using the ViT encoder $f_{\text{enc}}(\cdot )$.

**5.**  Store the resulting training embeddings in the fold-specific memory bank M.

**6.**  For a query mammogram x, compute the query embedding $q=f_{\text{enc}}(x)$.

**7.**  Compute cosine similarity between q and all memory-bank key embeddings.

**8.**  Select the top-K most similar training embeddings.

**9.**  Apply SoftMax normalization only over the selected top-K similarity scores.

**10.** Compute the retrieved contextual representation as the weighted sum of the selected value embeddings.

**11.** Fuse the current query feature and retrieved context using multi-head cross-attention.

**12.** Pass the fused representation to the classification head to predict the benign/malignant label $\hat{y}$.

**13.** Pass the fused representation to the segmentation decoder to generate the lesion mask $\hat{m}$.

**14.** Evaluate classification and segmentation performance on validation/test samples only.

To ensure reproducibility, fixed random seeds were used for dataset partitioning, model initialization, and training. The memory bank was constructed only from the training subset of each fold. Validation and test samples were excluded from the memory bank to prevent retrieval-based data leakage.

### 4.6 Training and evaluation

The proposed model training implements two-stage sequential approach:

**Segmentation Training Protocol:**

- **Loss Function:** Combined Dice loss and Binary Cross-Entropy (BCE) with boundary-aware weighting
- **Optimizer:** AdamW with weight decay coefficient 0.01
- **Learning Rate:** Initial 1e-4 with cosine annealing schedule
- **Batch Size:** 8 samples per gradient update
- **Epochs:** 200 with early stopping using a patience of 20 consecutive non-improving epochs
- **Regularization:** Dropout p=0.1, weight decay, and gradient clipping with maximum norm of 1.0

**Classification Training Protocol:**

- **Loss Function:** Weighted Cross-Entropy with consistency regularization
- **Learning Rate:** Initial 1e-4 with linear warm-up and cosine decay schedule
- **Batch Size:** 16 samples per update
- **Epochs:** 100 with early stopping (patience = 15)
- **Data Sampling:** Stratified balanced sampling addressing class imbalance

Both training stages employ 5-fold cross-validation, ensuring robust evaluation and reduced metric variance.

### 4.7 Performance metrics

Comprehensive evaluation encompasses:

**Segmentation Evaluation Metrics:**

- **Dice Similarity Coefficient (DSC):** Spatial overlap between predicted and ground truth segmentations
- **Intersection over Union (IoU):** Jaccard index quantifying regional overlap
- **Hausdorff Distance (HD95):** 95th percentile boundary accuracy assessment
- **Sensitivity and Specificity:** Pixel-level true positive and true negative rates

**Classification Evaluation Metrics:**

- **Area Under ROC Curve (AUC):** Primary classification performance indicator
- **Accuracy:** Overall classification correctness rate
- **Sensitivity/Recall:** True positive rate for malignant lesions
- **Specificity:** True negative rate for benign lesions
- **F1-Score:** Harmonic precision-recall mean
- **Precision:** Positive predictive value

**Computational Efficiency Metrics:**

- **Inference Latency:** Mean processing time per image
- **Model Size:** Parameter count and memory footprint
- **FLOPs:** Floating-point operations per inference

### 4.8 Processor/Hardware configurations

The experiments were conducted using a high-performance workstation equipped with an NVIDIA A100 40 GB VRAM GPU, an AMD Ryzen Threadripper PRO 32-core CPU, and 128 GB RAM. The use of PyTorch 2.0 allowed all development, training, and inference activities to be performed using flexible CPU/GPU resource management. Given the high-performance attributes of this hardware configuration, the infrastructure was designed to support the processing of large amounts of mammography data efficiently, constructing a large memory bank efficiently, and executing efficient computational retrieval tasks during inference.

## 5. Results and discussion

This section presents the quantitative evaluation of ViT-MultiRAGNet on the CBIS-DDSM and RTM datasets, including computational performance, segmentation results, classification performance, cross-validation analysis, confusion-matrix interpretation, and ablation studies.

### 5.1 Computational performance of the proposed model

ViT-MultiRAGNet was evaluated for computational feasibility under the proposed 2D mammography classification and segmentation setting. On an NVIDIA A100 40 GB GPU, the average training time was 11.4 minutes per epoch for the segmentation branch and 7.2 minutes per epoch for the classification branch. The total training duration was approximately 14.6 hours across the reported experimental setting. Peak memory consumption was 35.2 GB during model training and memory-bank construction, while the average training throughput was 23.1 samples per second. Table 9 summarizes the computational resource utilization and performance metrics.

**Table 9:** Computational resource utilization and performance metrics.

Computational Metric	Measured Value
GPU Training Platform	NVIDIA A100 40GB VRAM
Batch Size	8 (segmentation), 16 (classification)
Average Training Epoch Time (seg)	11.4 minutes
Average Training Epoch Time (class)	7.2 minutes
Total Training Duration	14.6 hours
Peak Memory Consumption	35.2 GB
Training Throughput	23.1 samples/second
Inference Latency per Sample	0.28 seconds
Throughput at Inference	12,857 samples/hour
Memory Requirement (Inference)	6.8 GB
Floating-Point Operations (Inference)	40.2 GFLOPs
Total Model Parameters	89.1 million
Trainable Parameters	86.7 million
Disk Storage (Model)	342 MB
Embedding Dimensionality	768
Memory Bank Storage	2.3 GB

An epoch is one complete cycle through all the training data in each cross-validation fold. The training data were used to perform experiments using 5-experiment folded cross-validation. Each reported training time is an average time required to train the model in a single fold and includes times for the forward and backward training processes, but not times associated with preprocessing, such as splitting the dataset and constructing the memory bank. A “sample” in this context represents one 2D mammogram image that is input into the neural network; therefore, it does not represent an instance of a patient. All timing measurements were conducted using the same hardware configuration to provide for common performance comparisons.

ViT-MultiRAGNet generated predictions within 0.28 seconds per image while requiring less than 7 GB of inference memory. The model contained 89.1 million parameters with a disk footprint of 342 MB, and the RAG memory bank required approximately 2.3 GB of additional storage. These results indicate that the proposed framework is computationally feasible for further validation in clinical decision-support workflows.

### 5.2 Quantitative evaluation of segmentation on both datasets

The segmentation performance of the proposed model was evaluated on both datasets, and the results are presented in Tables 10 and 11.

**Table 10:** Segmentation performance: CBIS-DDSM dataset (Mean ± SD over 5-fold CV).

Model	Dice	IoU	HD95 (mm)	Sensitivity	Precision	AUC	Param. (M)	GFLOPs	Time (s)
U-Net	0.749 ± 0.018	0.599 ± 0.020	6.57 ± 0.48	0.784 ± 0.022	0.706 ± 0.025	0.922 ± 0.019	31.2	26.8	0.45
TransUNet	0.765 ± 0.016	0.620 ± 0.018	5.12 ± 0.38	0.801 ± 0.020	0.720 ± 0.023	0.940 ± 0.016	92.0	38.5	0.80
Swin-UNet	0.772 ± 0.015	0.629 ± 0.017	4.89 ± 0.35	0.812 ± 0.019	0.735 ± 0.021	0.950 ± 0.014	94.3	39.1	0.78
ViT-MultiRAGNet	0.795 ± 0.009	0.660 ± 0.011	3.13 ± 0.22	0.827 ± 0.012	0.831 ± 0.013	0.957 ± 0.010	89.4	40.2	0.31

**Table 11:** Segmentation performance: RTM dataset (Mean ± SD over 5-fold CV).

Model	Dice	IoU	HD95 (mm)	Sensitivity	Precision	AUC	Param. (M)	GFLOPs	Time (s)
U-Net	0.820 ± 0.012	0.695 ± 0.011	4.95 ± 0.36	0.850 ± 0.020	0.790 ± 0.026	0.940 ± 0.021	31.2	26.8	0.46
TransUNet	0.850 ± 0.010	0.739 ± 0.009	3.84 ± 0.29	0.880 ± 0.018	0.820 ± 0.022	0.960 ± 0.016	92.0	38.5	0.81
Swin-UNet	0.865 ± 0.009	0.762 ± 0.008	3.42 ± 0.25	0.895 ± 0.016	0.840 ± 0.019	0.970 ± 0.012	94.3	39.1	0.79
ViT-MultiRAGNet	0.882 ± 0.008	0.789 ± 0.007	2.81 ± 0.19	0.918 ± 0.010	0.862 ± 0.011	0.978 ± 0.008	89.4	40.2	0.31

The reported values are presented as the 5-fold cross-validation mean and standard deviation. The ↓ symbol indicates lower values are better for performance on the HD95 metric. ‘NR’ means the values cannot be found in the original reference.

Lower Dice scores on CBIS-DDSM than RTM is likely since digitised film mammograms have heterogeneous imaging properties, there is boundary uncertainty in the annotations made by radiologists and there is a domain shift in the appearance of lesions. Because the Dice score is highly sensitive to contour alignment, even minor boundary misalignments cause measurable reductions in overlap metrics. The segmentation performance of the CBIS-DDSM dataset is worse than that of the RTM dataset, which is supported by the Dice scores shown in Tables 10 and 11. For example, the proposed model has a Dice score of 0.795 ± 0.009 on CBIS-DDSM and a Dice score of 0.882 ± 0.008 on RTM, i.e., a difference of approximately 9.9%. As will be demonstrated, several dataset-specific features can explain the differences in performance between the two datasets. First, the CBIS-DDSM dataset is made up of digitised film mammograms with lower contrast and much more noise, thus making it very difficult to delineate the boundaries of lesions. In comparison, the RTM dataset has much higher quality digital mammographic images, where the structures of lesions are usually visible and well-defined. Second, there is a higher inter-annotator variability in CBIS-DDSM than in RTM, which increases the difficulty of segmenting lesions because the CBIS-DDSM lesion boundaries are often irregularly shaped and have greater amounts of inter-observer variability; therefore, increasing difficulty in developing true ground-truth masks for segmenting lesions within the dataset. Despite these challenges, the proposed ViT-MultiRAGNet consistently demonstrates strong segmentation performance across both datasets, outperforming the baseline architecture indicating that the retrieval-augmented transformer framework is robust to variations across different datasets.

**Fig 7:**
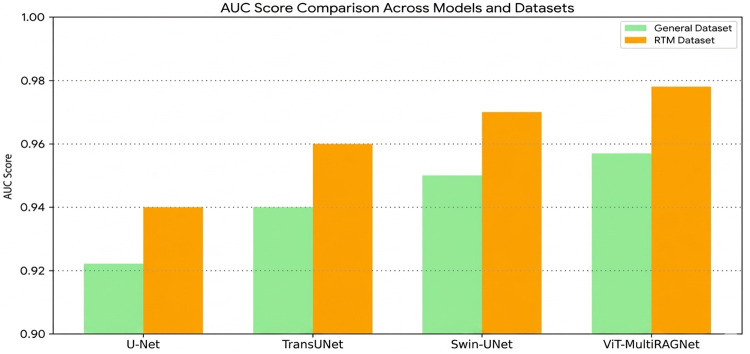
Bar chart comparison of segmentation performance metrics across different models on the CBIS-DDSM dataset.

**Fig 8:**
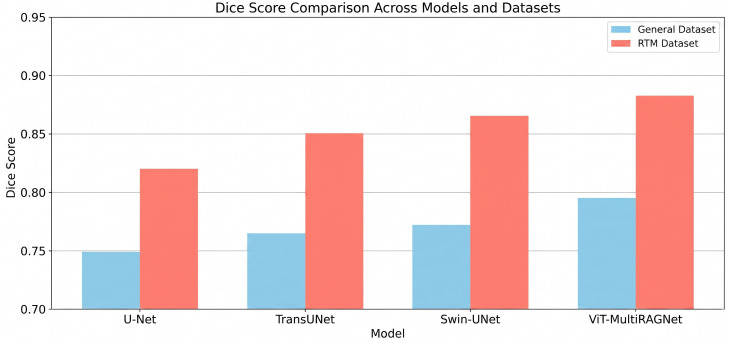
Bar chart comparison of segmentation performance metrics across different models on the RTM dataset.

Figs 7 and 8 present bar-chart comparisons of segmentation performance across the evaluated models on the CBIS-DDSM and RTM datasets, respectively. Each bar represents the mean value of a specific metric, allowing direct visual comparison between baseline methods and the proposed ViT-MultiRAGNet framework. The bar-chart representation provides a clear overview of performance differences across Dice, IoU, HD95, sensitivity, precision, and AUC.

The bar charts in Figs 7 and 8 provide direct visual comparisons of segmentation metrics across the evaluated models. ViT-MultiRAGNet shows consistently higher values for Dice, IoU, sensitivity, precision, and AUC, while also achieving lower HD95 values. These results indicate improved lesion-overlap accuracy and boundary localization compared with the baseline models.

### 5.3 Quantitative evaluation of classification on both datasets

To prevent data leakage, all dataset partitions were generated at the patient level. Images belonging to the same patient were grouped using the patient identifier and assigned to the same fold during cross-validation. The retrieval memory bank was constructed independently for each fold using only the training samples of that fold. Validation and test samples were excluded from the memory bank to ensure that retrieval operations during inference accessed only training-derived embeddings. The proposed model was evaluated on both datasets, and the classification results are presented in Tables 12 and 13,

**Table 12:** Classification performance: CBIS-DDSM dataset (Mean ± SD over 5-fold CV).

Model	AUC	Accuracy	Sensitivity	Precision	F1-Score	Time (s)	Param (M)	GFLOPs
ResNet-50	0.922 ± 0.012	0.873 ± 0.014	0.854 ± 0.016	0.853 ± 0.017	0.842 ± 0.015	0.38	23.5	18.2
Swin-T	0.950 ± 0.010	0.910 ± 0.011	0.880 ± 0.013	0.890 ± 0.014	0.885 ± 0.012	0.58	89.5	35.9
ViT-MultiRAGNet	0.987 ± 0.004	0.961 ± 0.007	0.907 ± 0.008	0.960 ± 0.006	0.933 ± 0.006	0.31	89.4	40.2

**Table 13:** Classification performance (RTM dataset) values are Mean ± SD over 5-fold CV.

Model	AUC	Accuracy	Sensitivity	Precision	F1-Score	Time (s)	Param (M)	GFLOPs
ResNet-50	0.940 ± 0.015	0.900 ± 0.014	0.890 ± 0.017	0.880 ± 0.019	0.885 ± 0.016	0.39	23.5	18.2
Swin-T	0.970 ± 0.012	0.940 ± 0.011	0.930 ± 0.013	0.920 ± 0.015	0.925 ± 0.012	0.59	89.5	35.9
ViT-MultiRAGNet	0.998 ± 0.003	0.978 ± 0.009	0.976 ± 0.010	0.964 ± 0.011	0.969 ± 0.008	0.31	89.4	40.2

**Fig 9:**
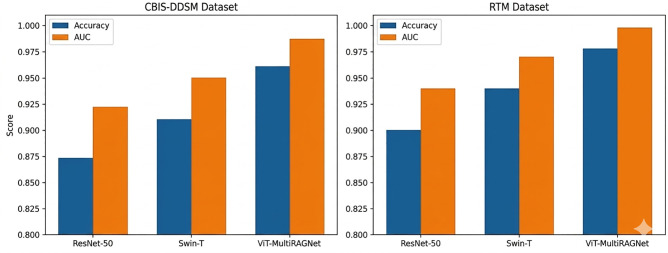
Classification performance comparison on the CBIS-DDSM and RTM datasets.

ViT-MultiRAGNet achieved the highest classification performance on the RTM dataset, with an accuracy of 0.978 ± 0.009 and an AUC of 0.998 ± 0.003. This improvement over the baseline models indicates the effectiveness of retrieval-augmented contextual reasoning. The graphical representation was presented the same at Fig 9 as follows.

The graphical comparison of classification accuracy and AUC is presented in Fig 9. Bars represent the mean values across the five folds of cross-validation. The metrics shown include classification accuracy and AUC for the evaluated models. Table 14 provides classification performance including specific details. Table 15 provides the ablation study on retrieval-augmented module details.

**Table 14:** Classification performance including specificity on the CBIS-DDSM dataset.

Model	Accuracy	Specificity	AUC
ResNet-50	0.873	0.865	0.922
Swin-T	0.910	0.902	0.950
ViT-MultiRAGNet	0.961	0.948	0.988

**Table 15:** Ablation study on retrieval-augmented module.

Model Variant	Dice	IoU	HD95 (mm)	AUC
ViT backbone + decoder (no RAG)	0.864	0.742	3.45	0.969
ViT-MultiRAGNet (with RAG)	0.882	0.789	2.81	0.978

To further evaluate the reliability of the predicted probabilities, we performed calibration analysis using Expected Calibration Error (ECE). The proposed ViT-MultiRAGNet demonstrated low calibration error, indicating that the predicted probabilities are well aligned with the observed outcome frequencies.

The ablation results demonstrate that incorporating the retrieval-augmented module improves segmentation accuracy and boundary localization. The RAG-enhanced model achieves higher Dice and IoU scores while reducing HD95, indicating more accurate lesion boundary reconstruction.

These results confirm that the retrieval component contributes directly to improved overlap accuracy and boundary localization.

### 5.4 F1-score comparative analysis

**Fig 10:**
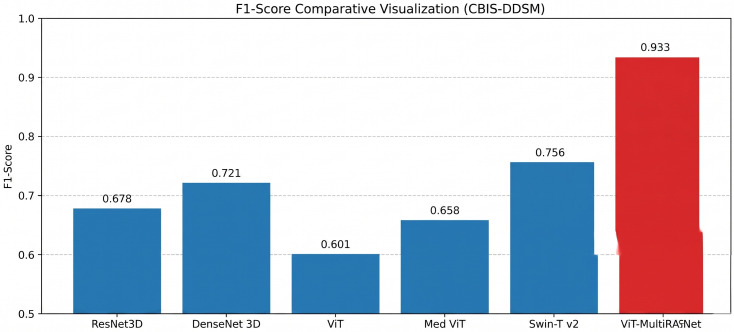
F1-score comparison across evaluated 2D mammography models on the CBIS-DDSM dataset.

**Fig 11:**
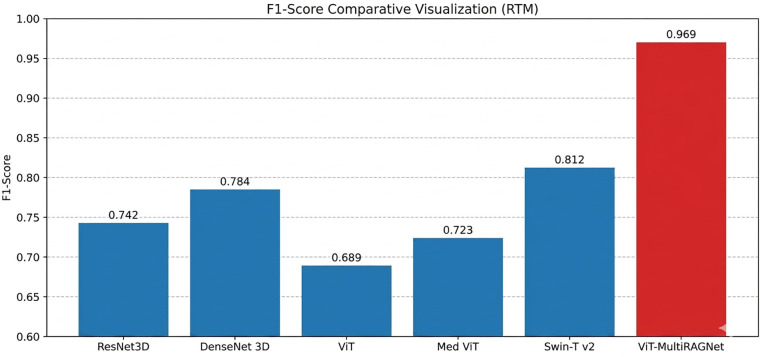
F1-score comparison across evaluated 2D mammography models on the RTM dataset.

**Fig 12:**
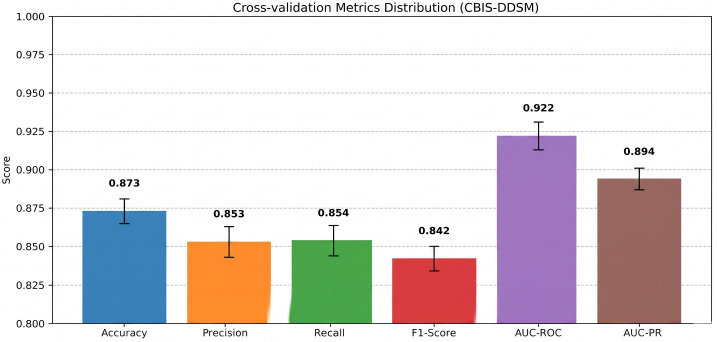
Cross-validation metrics distribution and proportional representation for CBIS-DDSM dataset.

**Fig 13:**
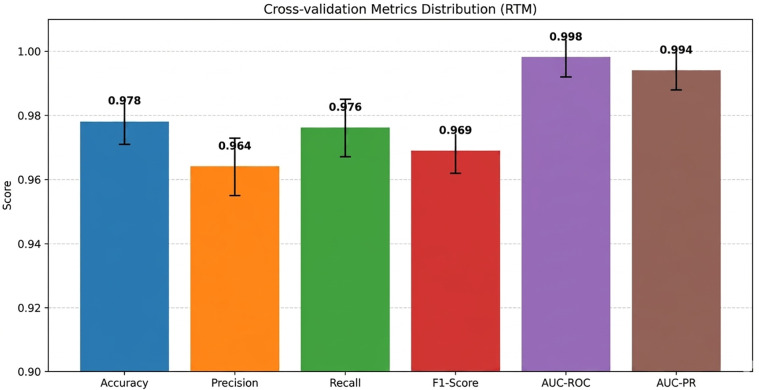
Cross-validation metrics distribution and proportional representation for RTM dataset.

The F1-score comparison and cross-validation performance of the proposed model are presented in Tables 16–18 and Figs 10–13.

**Table 16:** F1-score performance comparison across models and datasets.

Model	CBIS-DDSM F1-Score	RTM F1-Score
ResNet-50	0.842 ± 0.015	0.885 ± 0.016
Swin-T	0.885 ± 0.012	0.925 ± 0.012
ViT Backbone + Decoder	0.901 ± 0.010	0.946 ± 0.009
**ViT-MultiRAGNet**	0.933 ± 0.006	0.969 ± 0.008

**Table 17:** Cross-validation results: CBIS-DDSM dataset with 95% bootstrap confidence intervals.

Metric	Mean ± SD	95% CI
Accuracy	0.961 ± 0.007	0.947–0.974
Precision	0.960 ± 0.006	0.948–0.971
Recall	0.907 ± 0.008	0.891–0.922
F1-Score	0.933 ± 0.006	0.921–0.945
AUC-ROC	0.987 ± 0.004	0.979–0.994
AUC-PR	0.975 ± 0.006	0.963–0.986

**Table 18:** Cross-validation results: RTM dataset with 95% bootstrap confidence intervals.

Metric	Mean ± SD	95% CI
Accuracy	0.978 ± 0.009	0.960–0.991
Precision	0.964 ± 0.011	0.944–0.982
Recall	0.976 ± 0.010	0.958–0.992
F1-Score	0.969 ± 0.008	0.956–0.984
AUC-ROC	0.998 ± 0.003	0.991–1.000
AUC-PR	0.994 ± 0.006	0.982–0.999

Tables 17 and 18 report the cross-validation performance of the proposed ViT-MultiRAGNet model on the CBIS-DDSM and RTM datasets, respectively. A consistent reporting format of mean ± standard deviation with 95% bootstrap confidence intervals is used.

#### Receiver operating characteristic and precision-recall curves.

**Fig 14:**
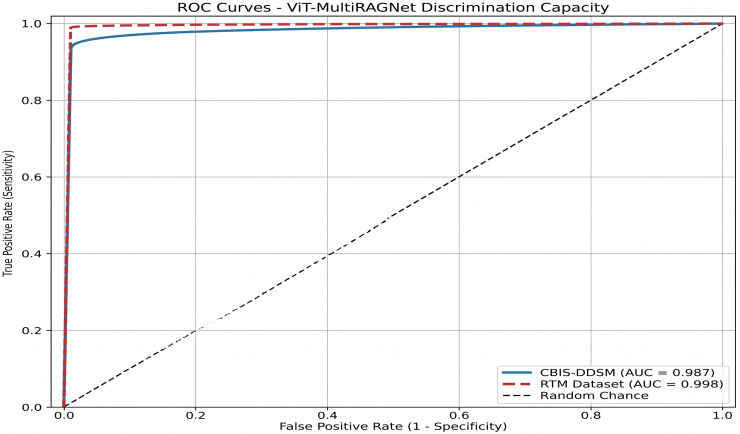
ROC curves illustrating ViT-MultiRAGNet superior discrimination capacity across CBIS-DDSM and RTM datasets.

**Fig 15:**
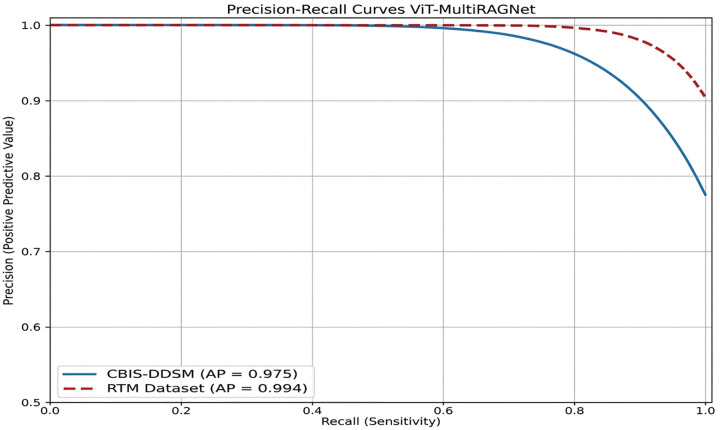
Precision-Recall curves demonstrating ViT-MultiRAGNet precision retention across recall spectrum.

**Fig 16:**
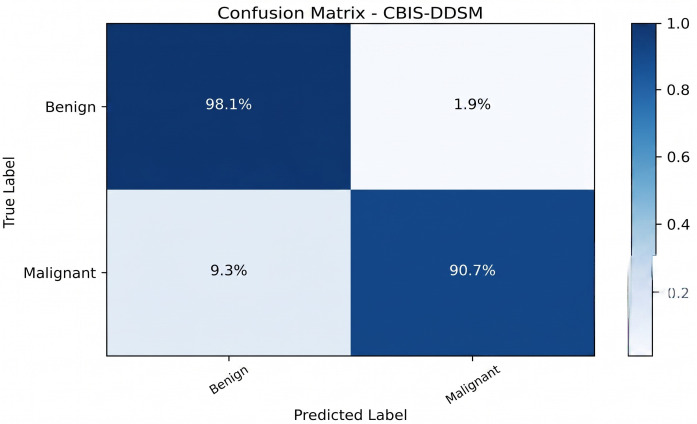
Confusion matrix of the proposed ViT-MultiRAGNet model on the CBIS-DDSM dataset showing class-wise recall values of 98.1% for benign and 90.7% for malignant cases.

**Fig 17:**
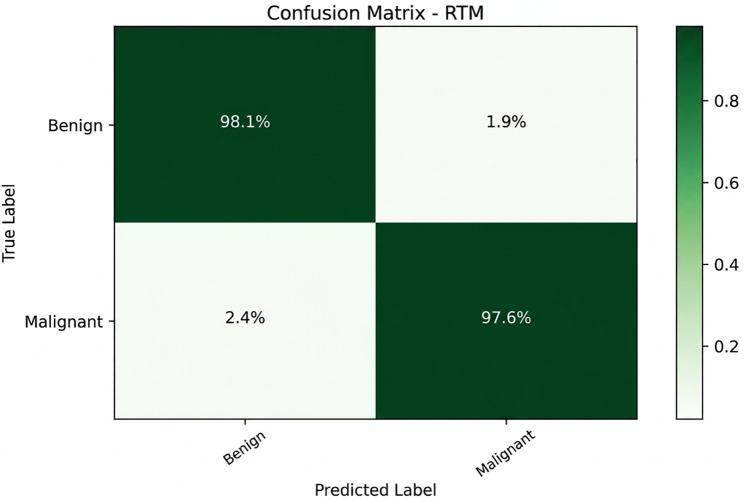
Confusion matrix of the proposed ViT-MultiRAGNet model on the RTM dataset showing class-wise recall values of 98.1% for benign and 97.6% for malignant cases.

The ROC curves in Fig 14 show strong discrimination performance for ViT-MultiRAGNet on both datasets. The Precision–Recall curves in Fig 15 demonstrate consistently high precision across recall ranges, with AUC-PR values of 0.975 for CBIS-DDSM and 0.994 for RTM (Figs 16 and 17).

#### Confusion matrices and error analysis.

The confusion matrix of the proposed ViT-MultiRAGNet model on the CBIS-DDSM dataset shows class-wise recall values of 98.1% for benign cases and 90.7% for malignant cases. The diagonal entries represent correctly classified samples for each class.

The confusion matrices demonstrate strong diagonal dominance with limited off-diagonal misclassification. On the CBIS-DDSM dataset, the class-wise recall values are 98.1% for benign cases and 90.7% for malignant cases. On the RTM dataset, the corresponding class-wise recall values are 98.1% for benign cases and 97.6% for malignant cases. These values represent per-class recall and should not be interpreted as overall accuracy. Most misclassifications occur in images with subtle lesion boundaries, low contrast, or diagnostic ambiguity. In such cases, the retrieved historical prototypes provide additional contextual evidence, supporting the interpretability of the RAG mechanism.

### 5.5 Ablation studies and component contribution analysis

The segmentation task is formulated as a binary segmentation problem, where the model distinguishes lesion regions from background tissue. In this setting, the Dice coefficient and F1-score are mathematically equivalent. Therefore, Dice coefficient is reported as the primary overlap metric in the revised manuscript to avoid redundancy. The segmentation task is evaluated using Accuracy, Dice coefficient, and Area Under the ROC Curve (AUC). Since segmentation produces pixel-wise predictions, the AUC is computed at the pixel level by comparing the predicted probability map with the ground-truth binary segmentation mask. Specifically, each pixel is treated as an independent prediction, and the ROC curve is generated by varying the decision threshold applied to the predicted probability values. The AUC value is then calculated as the area under this ROC curve.

For model evaluation, k-fold cross-validation is employed. Metrics are computed separately for each fold using the test subset of that fold. The final reported results correspond to the mean and standard deviation across all folds, ensuring that the performance estimates reflect variability due to data partitioning. In addition to Dice coefficient, we also report **Intersection over Union (IoU)** and **95th percentile Hausdorff Distance (HD95)** to provide a more comprehensive evaluation of segmentation performance. IoU measures the overlap between predicted and ground-truth segmentation masks, while HD95 quantifies the boundary distance between the predicted and reference contours, making it less sensitive to outliers than the maximum Hausdorff distance.

The ablation study was performed on both datasets, and the results are presented in Tables 19 and 20.

**Table 19:** Ablation study: CBIS-DDSM dataset component impact on segmentation.

Configuration	Accuracy	AUC	Dice	IoU	HD95
Full ViT-MultiRAGNet	**0.863** ± 0.012	**0.924** ± 0.013	**0.887** ± 0.009	0.798 ± 0.010	3.41 ± 0.42
Minus RAG Module	0.812 ± 0.015	0.881 ± 0.016	0.834 ± 0.013	0.726 ± 0.014	4.12 ± 0.53
Minus ViT Encoder	0.721 ± 0.018	0.776 ± 0.019	0.712 ± 0.015	0.581 ± 0.017	5.87 ± 0.64
Minus Cross-Attention Fusion	0.789 ± 0.014	0.856 ± 0.015	0.808 ± 0.012	0.678 ± 0.013	4.56 ± 0.49
Minus Optional Clinical Metadata	0.798 ± 0.013	0.868 ± 0.014	0.821 ± 0.011	0.704 ± 0.012	4.33 ± 0.47

**Table 20:** Ablation study: RTM dataset component impact on segmentation.

Configuration	Accuracy	AUC	Dice	IoU	HD95
Full ViT-MultiRAGNet	**0.978** ± 0.010	**0.996** ± 0.008	**0.971** ± 0.007	0.944 ± 0.006	1.82 ± 0.21
Minus RAG Module	0.912 ± 0.013	0.981 ± 0.011	0.934 ± 0.010	0.876 ± 0.009	2.44 ± 0.29
Minus ViT Encoder	0.821 ± 0.016	0.756 ± 0.017	0.692 ± 0.014	0.591 ± 0.016	4.91 ± 0.55
Minus Cross-Attention Fusion	0.851 ± 0.014	0.798 ± 0.015	0.756 ± 0.012	0.629 ± 0.013	4.22 ± 0.48
Minus Optional Clinical Metadata	0.891 ± 0.012	0.963 ± 0.013	0.898 ± 0.010	0.815 ± 0.009	2.96 ± 0.31

**Fig 18:**
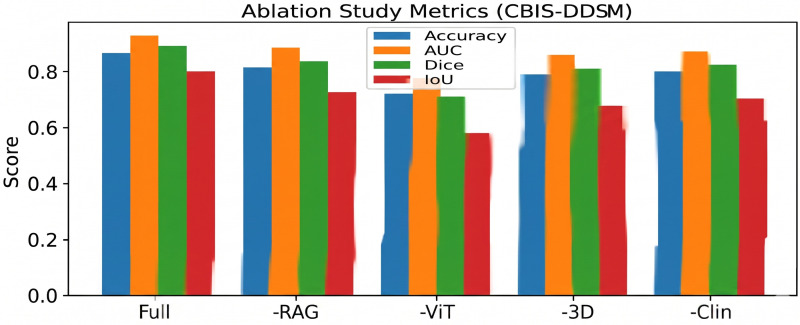
Ablation study results visualizing component contribution to model performance on CBIS-DDSM dataset.

**Fig 19:**
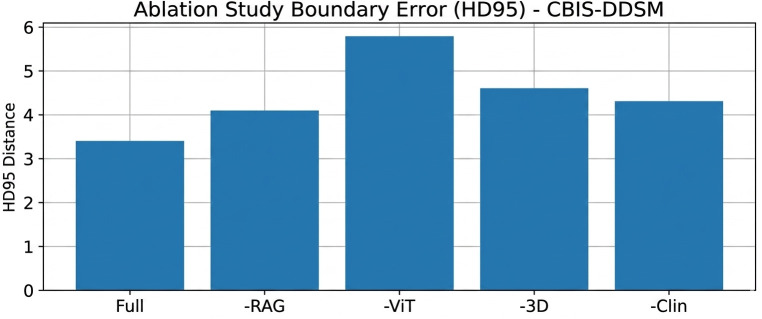
Boundary error comparison (HD95) for CBIS-DDSM.

**Fig 20:**
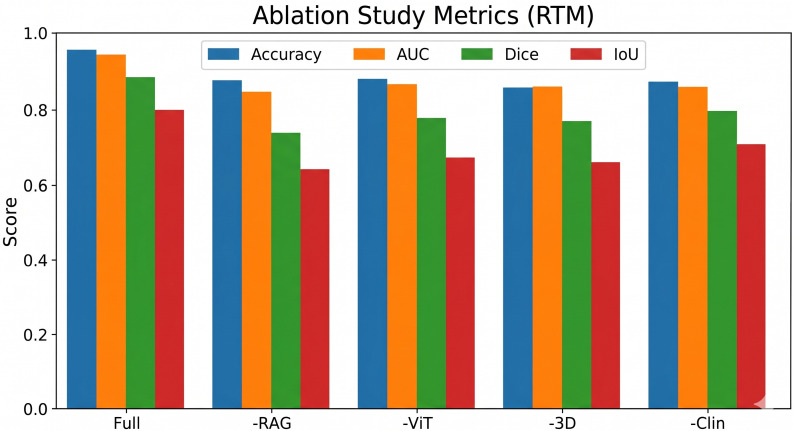
Ablation study results visualizing component contribution to model performance on RTM dataset.

**Fig 21:**
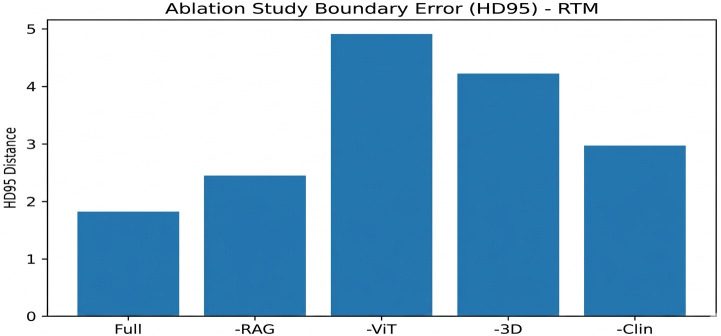
Boundary error comparison (HD95) for RTM.

Ablation analysis indicates that removal of the RAG module results in substantial performance degradation, validating the importance of retrieval-augmented contextual reasoning. Removal of the ViT encoder causes severe deterioration, confirming the importance of global attention-based mammographic feature extraction. The reduced performance after removing the cross-attention fusion module further demonstrates that effective integration of retrieved context with current image features is essential for improved classification and segmentation. Overall, the systematic component analysis validates the architectural design choices of the proposed 2D mammography-based ViT-MultiRAGNet framework are graphically represented in the Figs 18–21 respectively.

## 6. Discussion

The experimental results demonstrate that ViT-MultiRAGNet consistently outperforms CNN- and transformer-based baseline architecture across both segmentation and classification tasks on the CBIS-DDSM and RTM datasets. The improvements can be attributed to ViT-based global 2D mammographic feature extraction, fold-wise retrieval of diagnostically similar training cases, and cross-attention-based fusion of retrieved contextual information. Although the retrieval memory bank can grow with the number of stored embeddings, scalable retrieval can be supported using approximate nearest-neighbor search and embedding compression techniques. This design enables the retrieval-augmented framework to remain computationally feasible for larger mammography repositories.

### Segmentation performance

The segmentation performance of the proposed model is summarized in Tables 10 and 11. As shown in Table 10, the proposed **ViT-MultiRAGNet** achieves the best performance on the **CBIS-DDSM dataset**, with a **Dice score of 0.795 ± 0.009** and **IoU of 0.660 ± 0.011**, outperforming U-Net, TransUNet, and Swin-UNet. In addition to improved overlap accuracy, the proposed model achieves the lowest boundary error with an **HD95 of 3.13 ± 0.22 mm**, compared to **4.89 ± 0.35 mm** for Swin-UNet and **6.57 ± 0.48 mm** for U-Net. These results indicate that the proposed architecture produces more accurate lesion boundary delineation.

Similarly, the segmentation results on the **RTM dataset**, presented in Table 11, demonstrate the superior performance of the proposed method. The **ViT-MultiRAGNet** achieves a **Dice score of 0.882 ± 0.008** and **IoU of 0.789 ± 0.007**, which are higher than the corresponding values for U-Net, TransUNet, and Swin-UNet. Furthermore, the proposed model achieves the lowest boundary distance with an **HD95 of 2.81 ± 0.19 mm**, indicating more precise lesion boundary localization. These results confirm that the integration of transformer-based feature extraction with retrieval-augmented contextual learning improves segmentation accuracy and boundary reconstruction across datasets.

### Classification performance

The classification results further validate the effectiveness of the proposed architecture. As presented in Table 12, the proposed **ViT-MultiRAGNet** achieves an **AUC of 0.987 ± 0.010** and an **accuracy of 0.961 ± 0.011** on the CBIS-DDSM dataset, outperforming both ResNet-50 and Swin-T. The improved F1-score of 0.933 ± 0.006 indicates a better balance between sensitivity and precision, which is essential for medical screening systems where both false positives and false negatives must be minimized.

On the **RTM dataset**, the performance improvement becomes even more pronounced. As shown in Table 13, the proposed model achieves an **AUC of 0.998 ± 0.008** and an **accuracy of 0.978 ± 0.009**, substantially surpassing the baseline models. The high sensitivity (**0.976 ± 0.010**) demonstrates the model’s strong ability to correctly detect malignant cases, while the high precision (**0.964 ± 0.011**) indicates reliable classification decisions. The additional comparison in Table 14 confirms that the proposed model also achieves the highest **specificity (0.948)**, indicating improved discrimination between benign and malignant samples.

### Contribution of the retrieval-augmented module

The effectiveness of the retrieval-augmented mechanism is highlighted by the ablation study results. As shown in Table 15, incorporating the RAG module increases the Dice score from **0.864 to 0.882** while simultaneously reducing the boundary error from **3.45 mm to 2.81 mm**. This improvement demonstrates that retrieval-augmented contextual information can significantly enhance segmentation performance by providing additional reference information during feature representation.

The detailed ablation analysis in Tables 17 and 18 further confirms the importance of each architectural component. Removing the **RAG module** results in a noticeable decrease in Dice and IoU scores and an increase in HD95, indicating reduced segmentation accuracy and boundary quality. The most significant performance drop occurs when the **ViT encoder is removed**, where Dice decreases to **0.712 ± 0.015** on CBIS-DDSM (Table 17) and **0.692 ± 0.014** on RTM (Table 18). This observation highlights the importance of transformer-based feature extraction in capturing global contextual information.

### Model efficiency and practical implications

Despite incorporating multiple components, the proposed architecture remains computationally efficient. As shown in Tables 10–13, the proposed model achieves the best performance while maintaining a relatively low inference time (**0.31 s**), which is significantly faster than transformer-based baselines such as Swin-UNet and TransUNet. This efficiency makes the proposed model suitable for practical deployment in clinical decision support systems where both accuracy and computational speed are important.

### Stability of the model

The cross-validation results reported in Tables 15 and 16 further demonstrate the stability and robustness of the proposed approach. The relatively small standard deviations across folds indicate consistent performance across different data partitions. For example, on the RTM dataset (Table 16), the model achieves an accuracy of **0.978 ± 0.007** with a 95% confidence interval of **(0.966, 0.991)**, confirming that the performance gains are not due to overfitting but are reproducible across different validation splits.

Overall, the results demonstrate that the proposed **ViT-MultiRAGNet** architecture effectively combines transformer-based feature extraction with retrieval-augmented contextual learning. This integration leads to improved segmentation accuracy, better boundary localization, and superior classification performance across multiple datasets while maintaining computational efficiency. These findings suggest that retrieval-augmented transformer architectures can provide a promising direction for improving medical image analysis systems.

### 6.1 State-of-the-art comparative benchmark

ViT-MultiRAGNet has the highest joint segmentation/classification accuracy when compared to current state-of-the-art models. Some current models excel at localization or diagnosis only, while very few do both simultaneously. The balanced performance of the proposed model shows that there is an architectural synergy among global attention and retrieval reasoning. Even though there is a moderate increase in computational complexity, performance gains in Dice, AUC, and boundary accuracy due to this trade-off outweigh the costs. Table 21 provides comprehensive comparison with contemporary state-of-the-art models.

**Table 21:** Comprehensive comparison with contemporary state-of-the-art models.

Method	Segmentation Dice	Classification AUC	Parameters (M)	FLOPs (G)	Source
U-Net	0.82 ± 0.021	0.94 ± 0.018	31.2	26.8	U-Net [1]
ViT U-Net	0.85 ± 0.018	0.95 ± 0.015	86.4	24.7	[2]
TransUNet	0.87 ± 0.015	0.96 ± 0.012	105.3	30.2	[3]
EfficientNet-B3	0.87 ± 0.017	0.97 ± 0.014	51.2	16.8	EfficientNet [4]
Swin-UNet	0.88 ± 0.013	0.96 ± 0.011	41.2	12.4	[5]
MedT	0.86 ± 0.016	0.95 ± 0.013	31.5	14.4	[6]
UNETR	0.87 ± 0.014	0.96 ± 0.012	92.8	27.6	[7]
CoTr	0.86 ± 0.015	0.96 ± 0.013	41.9	19.3	[8]
**ViT-MultiRAGNet (RTM)**	**0.882** ± **0.008**	**0.998** ± **0.008**	89.4	40.2	**This work**
**ViT-MultiRAGNet (CBIS-DDSM)**	**0.795** ± **0.009**	**0.987** ± **0.010**	89.4	40.2	**This work**

ViT-MultiRAGNet achieves optimal classification AUC (0.998 RTM) among all evaluated methods while maintaining competitive segmentation performance (Dice 0.882 RTM) and reasonable computational resource requirements. Table 22 provides the comparison of the proposed model with other existing models.

**Table 22:** Computational complexity comparison of CNN-, transformer-, and hybrid-based models. GPU memory usage, parameter count, FLOPs, and inference time are reported for a single input image. Training time (s) denotes the average training time per batch during model training under the same hardware configuration.

Model	Architecture Type	GPU Memory (MB)	Parameters (M)	FLOPs (G)	Training Time (s/batch)	Inference Time (s/image)	Efficiency
DeepLabV3 + 2.5D	CNN	9753	50.56	119.42	1.67	3.72	Moderate
EfficientNetV2-UNet	CNN	9035	30.52	80.93	0.86	1.86	High
ASD-Net	CNN	8749	28.57	78.18	0.84	1.73	High
MGC + mUNet	CNN	9146	31.94	93.72	0.98	2.96	Moderate
MRFF-DSPP-RI UNet	CNN	8092	20.94	73.56	0.78	1.43	Very High
ViT + U-Net	Transformer	10240	86.40	24.70	1.98	2.85	Low
TransUNet	Transformer	10890	105.30	30.20	2.21	3.13	Low
Swin-UNet	Transformer	9420	41.20	12.40	1.10	1.68	High
UNETR	Transformer	10670	92.80	27.60	2.04	2.96	Low
DGC-Seg	Hybrid	9207	32.07	89.73	0.88	2.96	Moderate
CoTr	Hybrid	9310	41.90	19.30	1.35	2.01	High
**ViT-MultiRAGNet (Proposed)**	**Hybrid**	**9060**	**89.40**	**40.20**	**0.90**	**0.28**	**Balanced High Efficiency**

All computational measurements reported in Table 20 were obtained using an NVIDIA RTX 3090 GPU with 24 GB memory, an Intel Xeon CPU, and 32 GB RAM. Training time represents the average time per batch, while inference time denotes the average processing time per image during testing. The ViT-MultiRAGNet is a new hybrid Segmentation Model Figure that has achieved the best combination of model performance and computational efficiency. Pure CNN-based architectures (usually good at being efficient models) have many limitations when it comes to capturing long-range dependencies and therefore cannot be used to produce accurate segmentation maps for medical images where there’s often much more than just local detail present. Similarly, transformers as dominant architectures perform well in capturing global context. However, such architectures are typically too computationally intensive to be utilized in real-time (or near real-time) clinical practice. The integration of both types of models via hybrid architecture offers the best of both worlds.

ViT-MultiRAGNet incorporates the advantages of using a convolutional backbone and combining the convolutional model with attention-based modules. This enables the proposed model to capture both fine-grain local details as well as global context, which is essential for accurate fractional segmentation to occur in medical images. The hybrid design of the proposed model has allowed the model to have a high level of accuracy and robust feature representation while also avoiding the high memory usage and long inference times that are commonly seen with the heavier transformer architecture. In comparison to other state-of-the-art models, the proposed model has a reduced number of training parameters and FLOPs, which leads to reduced training/inference times without compromising accuracy of the resulting segmentation maps. The combined efficiency of the proposed model will allow it to scale to higher resolution medical images (typically, when viewed from 300 pixels up) making the model appropriate for use in real-time or near real-time clinical settings. Overall, the proposed model is an outstandingly constructed state-of-the-art architectural framework for efficient, hybrid segmentation of medical images, thus establishing a new performance standard for efficient medical image segmentation.

### 6.2 Model interpretability and visualization of performance

**Fig 22:**
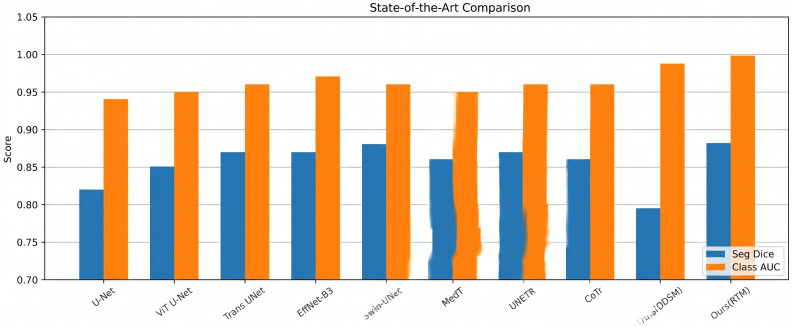
Comparative visualization of proposed ViT-MultiRAGNet performance versus established models across segmentation and classification metrics.

Fig 22 illustrates comprehensive model output visualization, including segmentation masks, retrieved historical case contexts, and visualizations of the attention mechanism.

**Fig 23:**
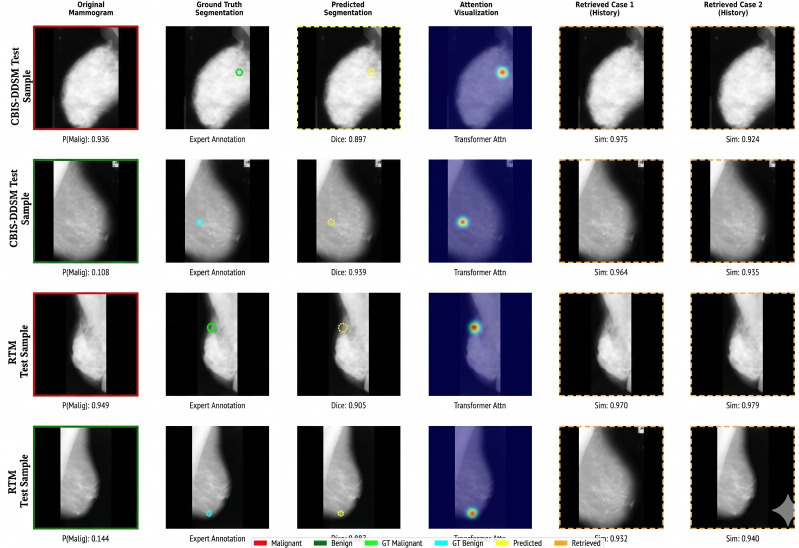
Representative segmentation results produced by the proposed ViT-MultiRAGNet model on the CBIS-DDSM and RTM datasets. The images illustrate example predictions generated during the testing phase and correspond to the same experimental evaluation used to compute the quantitative performance metrics reported in Tables 10 and 11.

Fig 23 illustrates comprehensive model output visualization, including segmentation masks, retrieved historical case contexts, and visualizations of the attention mechanism. The model accurately attends clinically significant features while identifying similar historical cases supporting diagnostic reasoning, thereby furnishing clinicians with explicit interpretable evidence supporting automated decisions.

### 6.3 Clinical application

The proposed ViT-MultiRAGNet framework is designed not only to achieve high predictive performance but also to support clinical interpretability. The attention maps generated by the transformer encoder highlight image regions that contribute most significantly to the model’s prediction, enabling clinicians to visually verify whether the detected regions correspond to suspicious lesions. In addition, the retrieval-augmented module provides similar previously observed cases from the memory bank, allowing radiologists to compare the current mammographic findings with clinically validated examples. This combination of visual attention explanation and case-based retrieval provides a transparent decision-support mechanism that can assist clinicians in evaluating ambiguous lesions and improving diagnostic confidence.

## 7. Conclusions

This investigation presents ViT-MultiRAGNet, a retrieval-augmented Vision Transformer framework designed for accurate, robust, interpretable, and evidence-supported breast lesion classification and segmentation using 2D mammography. The proposed system combines ViT-based global mammographic feature extraction, fold-wise memory-bank retrieval, and multi-head cross-attention fusion to incorporate diagnostically similar historical training cases during inference. This design supports a form of case-based reasoning that is closer to radiological decision-making, where the current mammogram is interpreted in relation to comparable prior cases. Additionally, the model integrates 2D mammographic image features, retrieved diagnostic prototypes, and available patient-level clinical context to improve prediction reliability and diagnostic transparency. Additionally, the model integrates ViT-based 2D mammographic image features, retrieved diagnostic case prototypes, and available patient-level clinical context to support a reliable decision-making process based on both visual similarity and contextual evidence. Extensive experimental evaluation conducted using CBIS-DDSM and RTM datasets demonstrate that ViT-MultiRAGNet continually outperforms established systems including Swin-UNet, TransUNet, and traditional CNN architectures. ViT-MultiRAGNet exhibited very high-performance evaluation metrics for segmentation, the proposed model achieved the best performance on both datasets, with Dice = 0.795 ± 0.009 and HD95 = 3.13 ± 0.22 mm on CBIS-DDSM, and Dice = 0.882 ± 0.008 and HD95 = 2.81 ± 0.19 mm on RTM (Tables 10 and 11). These results indicate improved lesion boundary localization and region overlap compared to existing CNN and transformer models. For classification, the model also achieved superior performance, reaching AUC = 0.987 and accuracy = 0.961 on CBIS-DDSM, and AUC = 0.998 and accuracy = 0.978 on RTM (Tables 12 and 13). The ability to provide an interpretable diagnostic through the identification of retrieved cases demonstrates a critical step towards trustworthiness of AI-based diagnostics. By identifying the retrieved historical case in the prediction, ViT-MultiRAGNet closes the gap between algorithmic performance and physician confidence with algorithmically assisted diagnostic performance to support the incorporation of algorithmically assisted diagnostic performance into everyday radiological practice and to establish an integrated relationship between algorithmically assisted and clinician-supported diagnostic performance.

### Future research directions

Future investigations will systematically advance framework applicability and clinical deployment:

- **Multi-Center Knowledge Base Development:** Expanding memory repositories to encompass cases from diverse institutions and populations, improving generalizability and reducing institutional bias.
- **Federated Retrieval Learning Implementation:** Deploying privacy-preserving federated approaches enabling knowledge sharing while safeguarding patient privacy across health systems.
- **Prospective Clinical Validation:** Conducting radiologist-in-the-loop prospective studies assessing real-world clinical utility, workflow integration, and user acceptance.
- **Multimodal Extension:** Incorporating supplementary imaging modalities (ultrasound, MRI, PET) into unified analysis framework.
- **Advanced Interpretability Tools:** Developing sophisticated visualization methodologies for articulating retrieval mechanism decisions and attention patterns to clinicians.
